# Advances in gene therapy for muscular dystrophies

**DOI:** 10.12688/f1000research.8735.1

**Published:** 2016-08-18

**Authors:** Hayder Abdul-Razak, Alberto Malerba, George Dickson

**Affiliations:** 1 School of Biological Sciences, Royal Holloway, University of London, Egham, Surrey, TW20 0EX, UK

## Abstract

Duchenne muscular dystrophy (DMD) is a recessive lethal inherited muscular dystrophy caused by mutations in the gene encoding dystrophin, a protein required for muscle fibre integrity. So far, many approaches have been tested from the traditional gene addition to newer advanced approaches based on manipulation of the cellular machinery either at the gene transcription, mRNA processing or translation levels. Unfortunately, despite all these efforts, no efficient treatments for DMD are currently available. In this review, we highlight the most advanced therapeutic strategies under investigation as potential DMD treatments.

## Introduction

Duchenne muscular dystrophy (DMD) is a rare, severe, degenerative X-linked myopathy caused by mutations in the gene ( *DMD*) encoding the dystrophin protein. DMD affects about 1 in 3500 to 4000 boys globally ^1, 2^, and one-third of cases is attributed to spontaneous new mutations ^3^. Dystrophin plays a key role in joining the actin of the cytoplasmic cytoskeleton to the extracellular matrix surrounding the sarcolemma of muscle fibres directly binding a protein complex known as dystrophin-associated glycoprotein complex (DGC) ^4^. Several proteins like alpha-dystrobrevin, syncoilin, synemin, sarcoglycan, dystroglycan, and sarcospan are located at the DGC, where they mediate the muscle-networking signals that are essential for both muscle cell function and the development and maintenance of membrane integrity ^2, 5^. Mutations in the *DMD* gene usually cause partial or complete absence of dystrophin, leading to membrane instability and muscle cell death associated with progressive tissue degeneration, muscle weakness, joint contractures, and kyphoscoliosis ^6, 7^. Both skeletal and cardiac muscles are affected in patients with DMD. In particular, the loss of dystrophin leads to respiratory failure ^8^ because of the severely damaged diaphragm and to cardiomyopathy and heart failure ^9^, which usually result in premature death by the age of 25 to 35 ^10, 11^, although some long-term survivors have been observed ^12^.

Despite more than 20 years of research and the clear understanding of the molecular basis of the disease, only limited advancement of therapeutic approaches has been obtained, and currently the only available treatment is a combination of physiotherapy and corticosteroids. Although these palliative treatments can provide a considerable improvement in affected boys, they can only slow the course of the disorder. In this article, we will discuss the relevance of three approaches for the treatment of DMD: the gene addition approach by triple trans-splicing (TTS), the use of gene editing to correct the *DMD* transcript, and the exon-skipping approach to re-frame the faulty *DMD* pre-RNA. All of these methods are promising DMD treatments that tackle the cause of the disease and offer the potential to treat many *DMD* mutations.

## Facing DMD challenges

The *DMD* gene is expressed mainly in skeletal and cardiac muscle. About 65% of the mutations affecting dystrophin are deletions of part of the gene with two predominant hotspots within the *DMD* sequence. Two types of mutations are associated with two allelic forms of the disease: the first leads to the expression of out-of-frame mRNA that abolishes dystrophin production and causes the most severe form-of-the-disease, the DMD. The second type of mutation induces the expression of an in-frame mRNA, resulting in a milder form of DMD known as Becker muscular dystrophy (BMD), in which a shorter but still partially functional dystrophin is produced ^13^.

There are currently many pharmacological approaches that can hinder DMD symptoms by focusing on secondary effects, yet they would treat just one aspect of DMD pathogenesis and may be associated with possible side effects ^14^. For a better understanding of the different potential therapies available for DMD, we firstly have to highlight which challenges they face. In this regard, one hurdle is that DMD is frequently caused by new mutations, meaning that many patients have no family history ^15^. Furthermore, more than 60% of *DMD* mutations are due to intragenic deletions of one or more exons, resulting in disruption of the correct *DMD* open reading frame (ORF) ^16^. Thus, DMD mutations can vary in severity and the phenotype is often unpredictable.

Therefore, the most urgent interest is to develop a genetic strategy that can provide a treatment for all patients with DMD. The other crucial point is that the ideal DMD therapy should be sustainable and lifelong. Previous observations in patients with X-linked myopathy suggest that in order to prevent muscle weakness, at least 30% of dystrophin must be expressed in skeletal muscle ^17^. Even if this may seem a low level compared with the one in normal tissues, a crucial issue is that muscles of boys with DMD are substantially wasted and present a significant amount of fat and connective tissue that makes the remaining muscle tissue hardly accessible by the therapeutic agents delivered to the bloodstream. Furthermore, although most therapeutic agents have proven to be successful in small animal models (for example, rodents), human muscles are significantly larger and this requires scaling up the therapeutic agents’ manufacturing process, which usually is related to logistic and economical challenges. Nowadays, gene therapy has emerged as a promising applicable strategy, as DMD therapy can cure the genetic defect and not just its downstream effects.

## *DMD* gene addition by trans-splicing

The vast majority of gene therapy-based clinical trials for other disorders are based on the gene addition “replacement” approach ^18^. For DMD gene replacement, a point of major concern is the size of the very long *DMD* gene (exonic DNA >11 kb). Hence, a partially functional, intact, and usually shortened *DMD* copy is delivered into muscle cells in order to mimic the beneficial effect of a smaller but partially functional protein expressed in patients with BMD. To achieve that, many delivery systems have been studied for DMD gene replacement, yet so far the most efficient is the use of adeno-associated virus (AAV) ^19– 21^. The main issue with AAV applications is that their limited packaging capacity of approximately 4.7 kb requires the delivery of substantially internally deleted (truncated) dystrophin expression cassettes. Additionally, when AAVs are used in large quantities, their capsid proteins could potentially activate an immunogenic response; consequently, strategies to attenuate the immune system are possibly needed for a clinically successful AAV approach ^3^.

In preclinical tests, single AAV vectors were used to successfully deliver microdystrophins (<5 kb)—carrying about 30% of the coding sequence, both locally and systematically—and were associated with good improvement of muscle function ^22– 25^. Nevertheless, some domains of dystrophin, like the critical parts of rod-and-hinge domains, should be included to increase dystrophin functionality and stabilise the membrane as they contribute to the recruitment of other components of DGC, such as neuronal nitric oxide synthase, syntrophin, and dystrobrevin ^26, 27^, on the sarcolemma. In order to expand AAV packaging capacity and improve muscle functionality, trans-splicing dual AAV vectors were developed about 15 years ago to deliver less than 10 kb minidystrophin ^28– 30^. In this approach, a large gene is divided, then packaged and delivered by two AAVs and ultimately expressed by dual trans-splicing. For this system to work, the first vector (the 5′ *DMD* part) is tail-tagged with splicing donor signal while the second vector (the 3′ *DMD* part) provides a preceding splicing acceptor signal. By co-transduction of the two vectors, the AAV head-to-tail vector genome concatemerisation ^31, 32^ and the removal of viral sequences from the transcribed mRNA by the cellular splicing machinery allow the expression of the entire minidystrophin protein ^33^. In two different studies, the localised injections of trans-splicing dual AAVs into a dystrophic muscle of a mouse model of DMD have shown successful minidystrophin expression in myofibres of a single muscle ^31, 34^. In another *in vivo* study, systemic injection led to high-level transduction within skeletal and cardiac muscles ^32^. Another possible strategy is based on dual protein trans-splicing that was applied in a mouse model leading to therapeutic gene expression and improving dystrophic muscle morphology and histology ^35^. Although the proof of principle for this approach was successfully demonstrated and muscle functions were slightly improved, transduction efficiency was too low to achieve the therapeutic level for a functional protein as minidystrophins still lack some potentially essential domains in the final dystrophin conformation.

This strategy may be improved by developing a system to deliver the full-length exonic *DMD* sequence and express an entirely functional dystrophin. In this regard, recent research has examined the possibility of dividing the whole native exonic *DMD* (~11 kb) into three AAV vectors, creating the Triple transplacing-AAV (TTS-AAV) system by using different inverted terminal repeats within the three vectors to favour the formation of the correct head-to-tail concatemers ^36^. The proof of concept was demonstrated in the DMD mouse model, showing the expression of a full-length dystrophin protein co-localising with the expression of a tagged protein (enhanced green fluorescent protein, or eGFP), whose coding sequence was at the 3′ end of the gene ^36^. However, the efficiency of the TTS-AAV system was very low. Only one out of four treated mice showed evidence of splicing between the three vectors. Further optimisation of this approach is essential to overcome the low efficient expression by, for example, improving splicing elements, maximising gene expression, and directing the AAV vector concatemerisation more efficiently. The TTS-AAV system then could bypass the issue of the AAV vector-related size limitation and pave the way for similar gene therapy applications involving large defective genes.

## *DMD* gene editing

Gene editing is the most exciting approach to treat DMD, as it harnesses the natural cellular repair mechanisms of non-homologous end joining (NHEJ) or homologous recombination (HR) to repair faulty genes at their endogenous loci. This approach would allow the gene to maintain its native regulation and to permanently correct the *DMD* defects. In somatic mammalian (including human) cells, the expected spontaneous NHEJ rate is approximately 1 in 10 ^3^ cells but for HR is approximately 1 in 10 ^6^ cells ^37, 38^. Notably, these low spontaneous frequencies have limited both the experimental and the therapeutic gene-editing strategies. Nevertheless, these rates could be increased via induction of site-specific DNA double-strand breaks (DSBs) by introducing custom designer nucleases to specifically cleave the DNA and then leave the cellular repair machinery to be recruited and correct the cleavage. In practice, engineered nucleases could be directed to target any defective gene within the genome by induction of site-specific DSBs that will be repaired by either NHEJ or HR. To date, nucleases such as meganucleases (MNs), zinc finger nucleases (ZFNs), transcription activator-like effector nucleases (TALENs), and clustered regularly interspaced short palindromic repeats (CRISPRs) systems have been tested in several models of disease.

The feasibility of designer nuclease-mediated gene editing has been shown in muscle disorders ^39– 43^. By applying MNs, the normal ORF of a dog microdystrophin containing a frame-shift mutation ^39^ was rescued while the expression of full-length dystrophin mRNA was restored in human patient myoblasts containing a deletion of exons 45–52 ^44^. This was the first example of successful genome editing of the DMD locus. ZFNs were used to target the dystrophin gene producing INDELs (insertion-deletions) of different sizes and leading to the restoration of the normal ORF ^40^. Recently, ZFNs were applied to successfully remove exon 51 from the dystrophin transcript, restoring the dystrophin normal ORF in cells of patients with DMD. This approach may lead to the treatment of approximately 13% of mutations of patients with DMD ^43^. The proof of principle of using TALENs for gene editing in primary dermal fibroblasts that originated from a DMD patient carrying a deletion of exons 46–50 was also recently published ^42^. Again, the *DMD* normal ORF was restored and functional dystrophin was ultimately expressed by targeting and removing exon 51.

It has lately been shown that AAVs can be used to successfully deliver CRISPR/Cas9 system into *mdx* mice to remove exon 23 from the dystrophin gene, leading to the expression of a partially functional dystrophin in skeletal myofibres and cardiac muscle, and also to improve muscle force ^45^. Moreover, it has recently been reported that TALENs and CRISPR/Cas9 systems can be applied side by side to correct the dystrophin gene in induced pluripotent stem cells derived from patients with DMD. In this case, the designer nucleases were used to disrupt the splicing acceptor to skip exon 45 of dystrophin, to induce small INDELs to correct the dystrophin ORF, and to knock in the exon 44 to restore full protein expression ^46^. In another study, TALENs and CRISPR nucleases were used to achieve a permanent restoration of *DMD* ORFs in patient-derived muscle cells. To achieve *DMD* restoration, short INDELs were incorporated at out-of-frame sequences to restore normal ORF, a splice acceptor was knocked out to skip exons permanently, and CRISPR-CRISPR or CRISPR-TALEN multiplexing was used to excise targeted exons ^47^.

Particularly important for a successful gene-editing approach in muscle disorders is to achieve and maintain a sustainable genetic correction by targeting both post-mitotic muscle tissue and muscle stem cells. Indeed, the correction of endogenous affected muscle stem cells would be advantageous, as their self-renewal feature would allow long-lasting regeneration of a patient’s muscles with newly formed corrected muscle cells ^48^. Despite promising results of applying designer nuclease-mediated gene editing for DMD treatment, there are still some crucial challenges: an efficient nuclease-based system should ensure a robust cleavage of the targeted DNA but for a very short time in order to achieve the desired site-specific DSBs and to control post-delivery nuclease expression at their target cells. Furthermore, possible related non-specificity and off-target cuttings (even at very low levels) are relevant concerns for the safety of this approach and preclude, at least with the currently available gene-editing tools, any *in vivo* application in humans.

## *DMD* exon skipping

The exon-skipping approach for DMD is based mainly on delivering antisense oligonucleotides (AONs) targeting some sequences associated with the exons and normally recognized by the cellular splicing machinery. AONs mask these sequences so that specific exons are spliced out from the pre-mRNA and the dystrophin ORF are restored by the expression of shortened BMD-like dystrophin with partial function ^49, 50^. In more than 80% of all DMD mutations ^13^ (including the majority of out-of-frame deletions ^51^), the skipping of one or two specific exons could lead to a correct *DMD* gene.

Since its first demonstration two decades ago ^52^, exon skipping has witnessed many optimisations and modifications in murine ^53– 55^ and canine ^56, 57^ models of DMD. Two AON chemistries have been used in clinical trial so far: 2′-O-methyl–modified ribose molecules with a full-length phosphorothioate backbone (2′OMePS) and phosphorodiamidate morpholino oligomers (PMOs). The largest group (13%) of all DMD patients could be treated by skipping exon 51 ^13^. The first clinical trial, based on intramuscular delivery of PRO051, a 2’OMePS AON, to induce exon 51 skipping was a success; sarcolemmal dystrophin was restored in 64 to 97% of examined myofibres ^58^. In subsequent phase II/III clinical trials, GlaxoSmithKline (London, UK) and BioMarin (formerly Prosensa) (Novato, CA, USA) tested the systemic delivery of the same AON, called drisapersen (latterly Kyndrisa). However, the results failed to demonstrate a statistically significant improvement in some crucial tests (for example, 6-minute-walk test) ^59^, and early this year, the US Food and Drug Administration (FDA) did not approve drisapersen as a marketable drug and this resulted in discontinuation of the drug ^60^. In parallel, AVI BioPharma (now known as Sarepta Therapeutics, Cambridge, MA, USA) developed AVI-4658 based on PMO chemistry to skip the exon 51 of dystrophin. After intramuscular injection into boys with DMD, AVI-4658 showed a 44 to 79% increase of dystrophin expression ^61^. The same AON tested systemically by Sarepta Therapeutics, renamed eteplirsen, increased dystrophin-positive fibres by 23% compared with placebo-injected controls ^62^. Eteplirsen then was systemically injected in a phase II/III clinical trial but with results similar to those obtained by the 2′OMePS-based AON drisapersen ^63^, and its approval by the FDA is currently under debate ^64^.

Despite promising results of systemic delivery of AONs for exon skipping, both in animal models and in humans, the treatment suffers from relatively poor efficiency. Increasing drug effectiveness by elevating dose levels over chronic time periods may not be feasible owing to the risk of toxic side effects. One other option is to increase the relative dose effectiveness without unduly exacerbating the risk of side effects. Thus, more research needs to be done to find better AON chemistries and possibly more efficient strategies to deliver them. New chemistries have recently been tested as tricyclo-DNA, a DNA analog, which was systematically administered into two DMD mouse models, leading to efficient dystrophin expression in both skeletal and cardiac muscles and, to a lesser extent, in the brain ^65^. Additionally, Gao *et al*. have reported that, in *mdx* mice, the repeated administration of peptide nucleic acid AONs, a synthetic chemistry, restores dystrophin in gastrocnemius, leading to amelioration of dystrophic pathology in DMD mice ^66^. Moreover, it has been demonstrated that systemic administration of PMO conjugated to cell-penetrating peptides resulted in high levels of dystrophin restoration in major respiratory muscles, including the diaphragm, and improved the cardiac function in *mdx* mice ^67^. Also, in an *mdx* model, it was recently demonstrated that dual exon skipping of dystrophin and myostatin pre-mRNAs using PMO conjugated with an arginine-rich peptide improved dystrophin expression and decreased muscle necrosis, particularly in the diaphragm ^68^. Likewise, the same principle was used to target dystrophin and Actvr2b to produce an internally deleted protein leading to comparable exon-skipping levels for both pre-mRNA targets when compared with individual PMO conjugates both *in vitro* and *in vivo* in *mdx* mice ^69^.

Alternatively, exon skipping of dystrophin exons could be achieved by using other approaches like CRISPR-Cas9 nuclease. In this regard, it has recently been shown that exon 23 could be skipped by using the CRISPR-Cas9 system when delivered to postnatal *mdx* mice intraperitoneally at postnatal day 1 (P1), intramuscularly at P12, and retro-orbitally at P18. Two CRISPR systems were used, preceding and following the mutated exon in the *DMD* gene, and delivered by AAV9. Following this genome editing treatment, restoration of dystrophin expression was detected at varied levels in both cardiac and skeletal muscles up to 12 weeks after injection ^54^. In a similar study, a dual CRISPR system was applied to induce a specific larger deletion across exons 50 and 54 of the *DMD* gene, resulting in fusion of the targeted exons. Whereas the *in vitro* experiments were performed in cultured 293T cells or DMD patient myoblasts (with deletion of exons 51–53), the *in vivo* experiments were done in the humanised hDMD/ *mdx* mice. Study results revealed that significant levels of hybrid exon 50/54 were formed *in vitro* in DMD myoblasts and that in hDMD/ *mdx* mice *in vivo* the corrected *DMD* ORF was partially restored ^55^.

The last two studies suggest that this gene-editing system may be advantageous over AON exon skipping, as a single injection leads to the permanent correction of the genome. However, no data on toxicity or off-target effects of using such gene-editing tools *in vivo* were reported. Those two studies highlight the importance of applying more than one approach to restore dystrophin. Indeed, a relevant amount of future research will focus on developing combined strategies to ameliorate the disease. As an example of this research direction, engineered CD133 ^+^ human DMD stem cells were transduced with lentivirus vectors that permanently delivered the cloned AONs rescuing murine dystrophin expression ^70^. Moreover, lately it was reported that co-administration of PMOs with glucose enhances exon-skipping activity in *mdx* mice ^53^.

Altogether, the *DMD* trans-splicing, gene-editing, and exon-skipping approaches, though still presenting some important issues, such as relatively low efficiency, possible associated toxic effects, or the need for chronic delivery, represent the most attractive and promising genetic treatments for DMD.

## Abbreviations

2′OMePS, 2′-O-methyl–modified ribose molecules with a full-length phosphorothioate backbone; AAV, adeno-associated virus; AON, antisense oligonucleotide; BMD, Becker muscular dystrophy; CRISPR, clustered regularly interspaced short palindromic repeat; DGC, dystrophin-associated glycoprotein complex; DMD, Duchenne muscular dystrophy; DSB, double-strand break; FDA, US Food and Drug Administration; HR, homologous recombination; INDEL, insertion-deletion; MN, meganuclease; NHEJ, non-homologous end joining; ORF, open reading frame; P, postnatal day; PMO, phosphorodiamidate morpholino oligomer; TALEN, transcription activator-like effector nuclease; TTS, triple trans-splicing; ZFN, zinc finger nuclease.
